# Can short-term heart rate variability predict coronary artery disease in patients undergoing elective coronary angiography due to typical chest pain?

**Published:** 2020-08-12

**Authors:** Ramaze Farouke Elhakeem, Mohamed Faisal Lutfi, Ahmed Babiker Mohamed Ali, Mohamed Yusif Sukkar

**Affiliations:** ^1^College of Medicine, Qassim University, KSA; ^2^Nile College of Medicine, Khartoum, Sudan; ^3^Faculty of Medicine and Health Sciences, Al-Neelain University, Khartoum, Sudan

**Keywords:** Autonomic modulation, Cardiac syndrome X, Sympathovagal balance

## Abstract

**Background:**

Presence of typical chest pain and normal coronary angiography suggests the possibility of microvascular ischemia of the myocardium as well as other non-cardiac causes that are also likely to decrease heart rate variability (HRV). This raises a question of whether poor HRV can predict abnormal elective coronary angiography (ECA).

**Aim:**

The aim of this study was to compare HRV in patients with typical chest pain when they are classified according to ECA outcomes.

**Methods:**

The study enrolled 150 patients planned for ECA in the cardiac center of AlShaab Teaching Hospital, Khartoum, Sudan, due to typical chest pain. Following assessment of medical history and clinical examination, the Bluetooth electrocardiography (ECG) transmitter and receiver were used for ECG recording and evaluation of time and frequency domains HRV. ECA confirmed the diagnosis of coronary artery disease (CAD) in 108 patients, who were considered as the test group. The other 42 subjects were considered as a control group after the exclusion of CAD.

**Results:**

The Mean±SD of Ln(pNN10), Ln(pNN20), LnLF, and LnHF was significantly higher in subjects with normal angiography compared with CAD patients. However, these statistically significant differences disappeared when the comparison was adjusted for age, gender, BMI, and HR of the studied groups.

**Conclusion:**

HRV is comparable in patients with typical chest pain regardless of ECA outcomes

**Relevance for patients:**

The HRV differences between patients with normal and abnormal ECA are likely to be biased by CAD risk factors such as old age, male gender, and tachycardia that are known to disturb HRV. The possibility of microvascular ischemia in patients with normal ECA may have attenuated HRV in this group and make it comparable to those suffering from macrovascular ischemia due to CAD.

## 1. Introduction

Assessment of heart rate variability (HRV) and cardiac autonomic modulation is important for risk stratification of heart diseases [1]. “NN” is commonly used instead of “RR” to stress that only normal heartbeats are processed during the evaluation of HRV [2]. HRV is a good indicator of morbidity and mortality associated with a wide spectrum of illnesses, including coronary artery disease (CAD) [3-5]. Poor HRV is linked to the hemodynamic derangements and risk stratification of CAD patients [6-8]. Alternatively, the presence of typical chest pain and normal coronary angiography suggests the possibility of microvascular ischemia of the myocardium [9] as well as other non-cardiac causes, for example, upper gastrointestinal causes [10], increased sensitivity to pain, anxiety [11], and other psychological factors [12]. Most of non-CAD causes of chest pain are also likely to induce low HRV [13,14]. This raises a question if poor HRV can predict abnormal elective coronary angiography (ECA). Although there are accumulating evidences of low HRV in CAD patients, researches exploring HRV measurements as predictors ECA are scare, if any. This study aimed to explore the possible HRV differences in patients with typical chest pain when classified according to ECA outcomes (normal vs. abnormal ECA).

## 2. Materials and Methods

The present study gained ethical clearance from the Ethics Review Committee (ERC), Faculty of Medicine, University of Khartoum, Sudan. All patients who agreed to join this study signed a written informed consent before being evaluated.

The study enrolled 150 patients planned for ECA due to typical chest pain. The study included all patients who agreed to join the study and is not known to suffer from congenital or acquired heart diseases during the period of data collection. All patients were seen on the same day intended for coronary catheterization in the cardiac center of AlShaab Teaching Hospital, Khartoum, Sudan. Medical history and clinical examination were performed to each subject guided by a questionnaire. A Bluetooth electrocardiography (ECG) transmitter and receiver (DM systems (Beijing) Co. limited – China) were used for ECG recording and evaluation of HRV. Five minutes ECG recording were started in each subject after ensuring the absence of artifacts on the ECG screen. ECG was performed in the supine position while breathing comfortably. Screening for various types of abnormal ECG recording was performed manually. Abnormal ECG readings such as ectopic beats, arrhythmias, and noise were deleted manually. Following visual inspection and manual editing, the software was allowed to calculate HRV parameters from the rest of the ECG data.

The studied time-domain HRV measurements were standard deviation of the normal to normal beat (NN intervals)), RMSSD (square root of the mean squared differences of successive NN intervals), and pNNx%. NNx is the number of pairs of successive NNs that differ by more than x ms. NNx was used to calculate pNNx using the following formula:


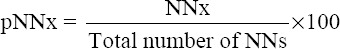


Bluetooth ECG transmitter and receiver software automatically calculate pNN10%, pNN20%, pNN30%, pNN40%, pNN50%, pNN60%, and pNN70% following the 5-min ECG recording. The studied frequency domain HRV measurements were total power (TP), very low frequency, low frequency (LF), high frequency (HF) power spectral densities, normalized low frequency (LF Norm), normalized high frequency (HF Norm), and LF/HF ratio. All HRV measurements were expressed by their natural logarithm (Ln).

LnSDNN and LnTP were used to evaluate overall HRV. LnRMSSD, Ln(pNNx), HF, and LnHF Norm to assess parasympathetic cardiac modulations, LnLF, and LF Norm to examine sympathetic cardiac modulations and Ln(LF/HF) to assess sympathovagal balance. The Bluetooth ECG transmitter and receiver software also provide the mean heart rate (HR) during the period of ECG recording (5 min).

ECA confirmed the diagnosis of CAD in 108 patients, who were considered as the test group. The diagnosis of CAD was made if ECA demonstrated one or more stenoses in ≥ half of the diameter of at least one major coronary artery, as described before [15,16]. The other 42 subjects were considered as a control group after the exclusion of CAD.

Statistical Package for the Social Sciences (SPSS) for Windows, version 16.0 (SPSS Inc., Chicago, IL, USA) was used for statistical analysis. The normal distribution of the studied variables was examined using Shapiro–Wilk test. Statistical differences between means of HRV were assessed using unpaired *t*-test. The age, gender, BMI, and HR were adjusted for while comparing HRV measurements between studied groups using the general linear model. *P*<0.05 was considered significant.

## 3. Results

Coronary artery catheterization of the studied subjects (*n*=150, m/f: 99/51) revealed 108 patients (m/f: 81/27) with CAD (% [95% CI]=72% [64.41-78.74%]) and 42 subjects (m/f: 18/24) with normal coronary arteries (28% [21.26-35.59%]). CAD patients had significantly increased HR, age, and akinesia/hypokinesia, but decreased BMI and ejection fraction (EF), compared with the control group, Table 1.

**Table 1:** Distribution of age, gender, BMI, HR, and MABP among the studied groups.

	Subjects with normal angiography *n*=42	Patients with abnormal angiography *n*=108	*P* value
Age, years (Mean±SD)	50.95±16.46	60.36±10.35	0.001
Male gender, % (95% CI)	42.86 (28.61-58.06)	75 (66.2-82.48)	<0.001
BMI, kg/m^2^ (Mean±SD)	29.61±5.06	26.29±4.55	0.001
HR, beat/min (Mean±SD)	68.99±10.76	76.28±27.11	0.020
MABP, mmHg (Mean±SD)	97.14±12.82	95.24±14.13	0.441
Hypertension, % (95% CI)	42.86 (29.12-57.79)	49.07 (39.84-58.37)	0.494
Diabetes mellitus, % (95% CI)	19.05 (9.982-33.3)	47.22 (38.07-56.57)	0.002
EF, (%)	59.34±9.63	52.08±12.78	0.006
Akinesia/hypokinesia, % (95% CI)	19.35 (9.19-36.28)	57.14 (46.01-67.6)	<0.001

BMI: Body mass index, HR: Heart rate, MABP: Mean arterial blood pressure, EF: Ejection fraction

The distribution of coronary arteries affected in the test group is given in Table 2.

**Table 2:** Distribution of coronary arteries affected in patients with CAD.

	% (95% CI)
LCA	18.52 (12.32-26.88)
LAD and its branches	91.67 (84.92-95.55)
Cx and its branches	57.41 (47.99-66.32)
Right main coronary artery	56.48 (47.07-65.45)
PDA and its branches	6.48 (3.178-12.78)

CAD: Coronary artery disease, LCA: Left main coronary artery, LAD: Left anterior descending, Cx: Circumflex, PDA: Posterior descending artery

The Mean±SD of Ln(pNN10), Ln(pNN20), LnLF, and LnHF was significantly higher in subjects with normal angiography compared with CAD patients, Table 3. However, these statistically significant differences disappeared when the comparison was adjusted for age, gender, BMI, and HR of the studied groups, Table 3.

**Table 3:** Comparison of HRV measurements between the studied groups.

	Subjects with normal angiography *n*=42 Mean±SD	Patients with abnormal angiography *n*=108 Mean±SD	*P* value	

			Non-adjusted	Adjusted*
LnSDNN	4.36±0.58	4.30±0.83	0.663	0.378
LnRMSSD	4.58±0.72	4.49±0.97	0.564	0.302
Ln(pNN10)	3.91±0.70	3.53±0.94	0.016	0.432
Ln(pNN20)	3.24±1.12	2.72±1.34	0.019	0.507
Ln(pNN30)	2.64±1.39	2.33±1.35	0.242	0.284
Ln(pNN40)	2.23±1.45	2.00±1.35	0.409	0.551
Ln(pNN50)	1.91±1.42	1.71±1.35	0.465	0.868
Ln(pNN60)	1.64±1.38	1.53±1.32	0.678	0.977
Ln(pNN70)	1.42±1.37	1.48±1.28	0.823	0.487
LnTP	6.39±0.92	6.04±1.17	0.057	0.481
LnVLF	5.65±0.90	5.33±1.18	0.114	0.391
LnLF	4.81±1.07	4.31±1.41	0.019	0.428
LnHF	4.68±1.25	4.18±1.46	0.041	0.593
LF Norm	50.05±18.35	48.78±20.15	0.712	0.629
HF Norm	43.55±16.74	42.23±17.16	0.668	0.853
Ln(LF/HF)	0.13±0.83	0.12±0.98	0.928	0.778

* Adjusted for age, gender, BMI, and HR. HRV: Heart rate variability, BMI: Body mass index, HR: Heart rate

**Figure 1:**
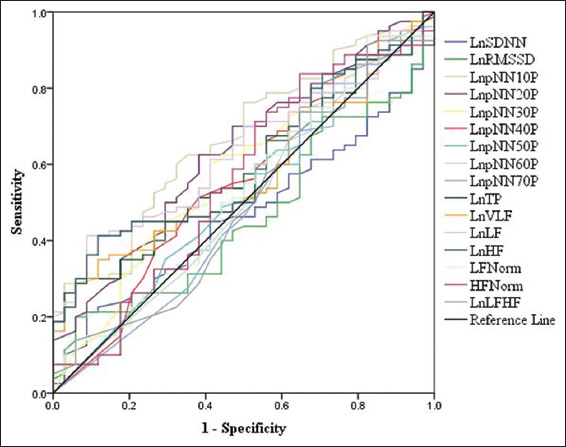
Comparison between receiver operating characteristic curves of heart rate variability measurement when used to predict elective coronary angiography outcome.

Using receiver operating characteristic (ROC) curve analysis, none of the HRV measurement achieved area under the curve (AUC) of more than 67 when used to predict ECA outcome, Figure 1, and Table 4.

**Table 4:** Comparison between AUC of HRV measurements when used to predict ECA outcome.

	AUC (95% CI)	*P* value
LnSDNN	0.47 (0.36-0.58)	0.584
LnRMSSD	0.46 (0.35-0.57)	0.528
Ln(pNN10)	0.67 (0.56-0.77)	0.005
Ln(pNN20)	0.63 (0.52-0.74)	0.026
Ln(pNN30)	0.58 (0.47-0.70)	0.157
Ln(pNN40)	0.55 (0.44-0.67)	0.363
Ln(pNN50)	0.53 (0.41-0.65)	0.644
Ln(pNN60)	0.50 (0.39-0.62)	0.941
Ln(pNN70)	0.48 (0.36-0.60)	0.743
LnTP	0.59 (0.48-0.70)	0.132
LnVLF	0.57 (0.47-0.68)	0.209
LnLF	0.63 (0.53-0.74)	0.025
LnHF	0.60 (0.50-0.71)	0.078
LFNorm	0.52 (0.40-0.64)	0.715
HFNorm	0.56 (0.44-0.68)	0.350
Ln(LF/HF)	0.49 (0.38-0.61)	0.909

AUC: Area under the curve, HRV: Heart rate variability, ECA: Elective coronary angiography

## 4. Discussion

The present study is probably the first report that explores the possible HRV differences in patients with typical anginal pain when classified according to ECA outcomes. Although the results of the present study reveal higher HRV in patients with normal coronary arteries compared to those with CAD, only LnpNN10%, LnpNN20%, LnLF, and LnHF achieved statistical significance. Following adjustment for age, gender, BMI, and HR, all measured HRV indices were comparable in patients with normal and abnormal coronary angiography. For further verification, the reliability of HRV measurements to predict ECA outcome was assessed using AUC and ROC curves. None of AUC was above 67, which indicates poor (70 > AUC > 60) or failure (AUC < 60) of HRV measurements to predict ECA outcome. The present findings should not be interpreted that those with normal ECA are healthy since they were suffering from typical chest pain. Recent reports on typical chest pain and normal coronary angiography suggest the possibility of cardiac syndrome X (CSX), a disease caused by microvascular ischemia of the myocardium.

Although the findings of the present study agree with some recent reports [8,17], other studies confirmed significantly higher HRV in healthy controls compared with CAD patients[18,19]. A recent study designed by Neves *et al*. compared autonomic modulations of healthy controls to CAD patients with and without acute myocardial infarction based on HRV measurements [8]. Unexpectedly, results revealed no significant differences in HRV parameters between both groups of CAD patients and healthy controls. Neves *et al.*, findings are further supported by another study which confirmed no differences in the frequency domain HRV when CAD and CSX patients were compared [17]. Frøbert *et al*. were able to prove low HRV in CSX patients with positive exercise ECG. In contrast, CSX patients studied by Frøbert *et al*. who had negative exercise ECG were comparable to the healthy control [20]. Other researchers were able to demonstrate low HRV in CSX patients during, but not in between myocardial ischemic episodes [21,22].

The results of the present study disagree with Kotecha *et al*. who declared that low HRV is strongly predictive of angiographically defined CAD, regardless of other comorbidities [23]. Simula *et al*. performed quantitative coronary angiography in 30 subjects without a history of myocardial ischemia but with high familial risk for CAD [6]. Coronary angiography of the studied subjects revealed mild stenosis of one or more of the main coronary vessels with means percentage of narrowing ranging between 25% and 35%. A negative correlation was documented between pNN50 and coronary artery stenosis. In addition, there was an inverse relationship between the power of HF spectral component and severity of coronary atherosclerosis. This fact suggests that the extent of coronary atherosclerosis is related to the change of cardiac autonomic modulation toward vagal withdrawal and sympathetic predominance even in subjects without evidence of myocardial ischemia. Comparable findings were demonstrated in Pivatelli *et al*. study, which showed significantly lower HF, pNN50, SDNN, and RMSSD in patients presented CAD [7].

Based on the current results, it appears that the observed differences in HRV between patients with normal and abnormal ECA are secondary to certain CAD risk factors that are known to disturb HRV, for example, old age, male gender, and tachycardia. Alternatively, the presence of microvascular ischemia in patients with typical chest pain and normal ECA may have attenuated HRV in this group and make it comparable to those suffering CAD. HRV measurements are, therefore, inappropriate predictors of abnormal ECA.

## 5. Conclusion

Although the results of the present study demonstrated higher HRV in patients with normal coronary arteries compared to those with CAD; only LnpNN10%, LnpNN20%, and LnLF achieved statistically significant differences. Following adjustment for possible confounders that are known to affect HRV, for example, age, gender, BMI, and HR, all measured HRV indices were comparable in patients with normal and abnormal ECA. Based on the present finding, it seems logical that the apparent differences in HRV between patients with normal and abnormal ECA are secondary to the CAD risk factors such as old age, male gender, and tachycardia that are known to disturb HRV. Alternatively, the presence of microvascular ischemia in patients with normal ECA may have attenuated HRV in this group and make it comparable to those suffering from macrovascular ischemia due to CAD. HRV measurements are, therefore, inappropriate predictors of abnormal ECA.

## Abbreviations

BMI body mass index; CAD coronary artery disease; CSX cardiac syndrome X; ECA elective coronary angiography; ECG electrocardiography; ERC ethics review committee; HF high frequency; HF Norm normalized high frequency; HR heart rate; HRV heart rate variability; LF low frequency; LF Norm normalized low frequency; Ln natural logarithm; NNx is the number of pairs of successive NNs that differ by more than x ms; pNNx% the percentage of the number of pairs of successive NNs that differ by more than x ms (NNx) out of total number of NN; RMSSD square root of the mean squared differences of successive NN intervals; SDNN standard deviation of the normal to normal beat; SPSS Statistical package for the social sciences; TP total power; VLF very low frequency.
